# Building theories of knowledge translation interventions: Use the entire menu of constructs

**DOI:** 10.1186/1748-5908-7-114

**Published:** 2012-11-22

**Authors:** Jamie C Brehaut, Kevin W Eva

**Affiliations:** 1 Ottawa Hospital Research Institute, Clinical Epidemiology Program, The Ottawa Hospital, General Campus, 501 Smyth Road, Centre for Practice Changing Research, Box 201B, Ottawa, ON K1H 8L6, Canada; 2 Department of Epidemiology and Community Medicine, University of Ottawa, Ottawa, ON K1H 8M5, Canada; 3 Centre for Health Education Scholarship, Department of Medicine, University of British Columbia, Vancouver, BC V5Z 4E3, Canada

**Keywords:** Theory, Audit and feedback, Knowledge translation, Constructs, Domains, Cognitive psychology, Education

## Abstract

**Background:**

In the ongoing effort to develop and advance the science of knowledge translation (KT), an important question has emerged around how theory should inform the development of KT interventions.

**Discussion:**

Efforts to employ theory to better understand and improve KT interventions have until recently mostly involved examining whether existing theories can be usefully applied to the KT context in question. In contrast to this general theory application approach, we propose a ‘menu of constructs’ approach, where individual constructs from any number of theories may be used to construct a new theory. By considering the entire menu of available constructs, rather than limiting choice to the broader level of theories, we can leverage knowledge from theories that would never on their own provide a complete picture of a KT intervention, but that nevertheless describe components or mechanisms relevant to it. We can also avoid being forced to adopt every construct from a particular theory in a one-size-fits-all manner, and instead tailor theory application efforts to the specifics of the situation. Using audit and feedback as an example KT intervention strategy, we describe a variety of constructs (two modes of reasoning, cognitive dissonance, feed forward, desirable difficulties and cognitive load, communities of practice, and adaptive expertise) from cognitive and educational psychology that make concrete suggestions about ways to improve this class of intervention.

**Summary:**

The ‘menu of constructs’ notion suggests an approach whereby a wider range of theoretical constructs, including constructs from cognitive theories with scope that makes the immediate application to the new context challenging, may be employed to facilitate development of more effective KT interventions.

## Background

Healthcare research costs over 100 billion dollars annually in North America alone [1,2]. This considerable investment yields important new knowledge that can significantly improve the health of patients and populations, provided that the knowledge is implemented appropriately. Over the last 20 years or so, it has become increasingly apparent that ‘appropriate implementation’ is an extraordinarily complex and multifactorial problem [3]. The techniques people have used to implement new knowledge have most often lacked any substantive justification, and have instead been based on past practice and logistical constraints rather than any in-depth understanding of what is likely to work. Perhaps not surprisingly, systematic reviews of many commonly used implementation techniques [4-8] have shown their effectiveness to be highly variable. While these realizations have coalesced into the science of implementation, also referred to as knowledge translation (KT), much remains to be understood about why strategies aimed at improving the use of new research knowledge to improve healthcare have proven so inconsistent in effect.

An important debate in this developing discipline pertains to the use of theory to understand the techniques and processes underlying KT. Some have argued that KT interventions are too heterogeneous for anything to be gained by trying to develop a generalizable theory [9,10]. More recently, however, the literature has largely subscribed to the arguments put forward by Eccles and others [3,11] that the benefits to be derived from theory in terms of generalizability of findings and standardization of methodology outweigh any risks.

Early efforts to understand KT techniques and processes have focused on identifying which existing social and health psychological theories can most successfully be applied in new KT contexts [12]. This approach correctly assumes that the psychological principles underlying one area of human endeavor often transfer to others [13] and, as such, may provide a head start towards identifying causal mechanisms that determine the effectiveness of the KT intervention in question [3]. Using existing theory (*i.e.*, ‘a coherent and non-contradictory set of statements, concepts, or ideas that organises, predicts, and explains phenomena, events, behavior, etc.’ [3,14]) has the further benefits of a foundation of both empirical work performed in other contexts and methodological innovations establishing strategies for measuring relevant constructs (*i.e.*, the abstracted concepts or explanatory variables on which theory is built) [3].

The literature has now seen the application of a variety of theories to various KT endeavors (see Godin [15] for a review). Based on this work, a number of lessons can be identified. First, the number of theories potentially available for study is enormous [16,17]. Second, theory may guide implementation in many ways [18], ranging from closely directing the development and implementation of the intervention, to crudely being used as a crutch to justify an intervention *post hoc*[19]. Third, considering theory alone, without empirical observation to identify the influence of a specific context, can miss important information relevant to improving KT [9,12]. Fourth, theories can differ in the roles they are intended to serve, with some clearly designed to provide explanation and prediction and others being intended primarily to serve communicative roles, a fact that can complicate application to new contexts [20]. In part because of these challenges, the majority of theory application work has focused on a very small number of theories [15] and yielded only modest progress on issues about how to improve KT interventions [21]. The best example of this is the Theory of Planned Behavior (TPB) [21,22], which has been attractive to KT researchers because of its broad scope (*i.e.*, it is explicitly intended to be applicable to all voluntary human behaviors) and its demonstrated applicability to a wide range of behaviors. Unfortunately, the TPB has proven less useful for generating clear recommendations for improving KT.

In response to these issues, some have called for an increased focus on theory construction in KT research, rather than the wholesale application of existing theories to contexts that may extend beyond their original mandate. For example, Rycroft-Malone [12] argues that development of context-specific ‘micro-theories’ would result in a better understanding of the KT issues that are specific to different stakeholders, disciplines, and settings. This might develop important localized information, but the inevitable risks of coarse application of such an approach would include lack of generalizability of findings and the necessity for considerable methodological groundwork for each new context.

We propose that there is a middle ground that might be usefully explored. Construction (as opposed to application) of a theory need not involve *de novo*, start-from-scratch theory building. Instead, when seeking causal mechanisms that contribute to successful KT, one could combine theory-building activities with individual constructs from any number of relevant theories. We will refer to this as the ‘menu of constructs’ approach, and argue that researchers looking to explain an area of KT should consider the entire menu, not just constructs associated with a particular theory.

We see the menu of constructs approach as attractive for a number of reasons. It allows for inclusion of only those constructs that are relevant to the new context, rather than requiring transport of the entire theory. Very often, it is the individual constructs, rather than the theory as a whole, that recommends the theory in the first place. For example, the notion that channel factors (*i.e.*, features of the environment that lead individuals to act in particular ways) will influence a clinician’s behavior may be more readily applied to KT interventions than the entire social psychological theory of situationism (*i.e.*, the notion that the situation in which one finds themselves is the dominant factor in determining an individual’s behavior [23]) in which channel factors are embedded. Theory newly constructed from a menu of constructs can: leverage measurement/methodological advances from the domain in which the various constructs were generated; incorporate both theory-based constructs and components of the specific context and behavior [24]; and propose KT interventions that are rooted in, but not restricted by, the larger body of theoretical literature.

The goal of this paper is to spur debate about the range of roles that theory (and theory-relevant constructs) should play in the overall endeavor to improve KT interventions. Our central claim is that theory development may progress more quickly if we allow ourselves to incorporate constructs derived from a range of theories, rather than feeling restricted to align/justify/use any particular theory in its entirety, thus broadening and tailoring the conceptual underpinning of specific KT interventions. In the following sections, we make our arguments in the context of audit and feedback (A&F) as an example KT intervention, but believe that the general logic will apply more broadly. We begin by explaining why we have chosen A&F as our example. We then provide some examples about how the menu of constructs approach is already being explored. We then discuss several examples of constructs that may inform our understanding of KT interventions, but have not been considered in the context of a theory of A&F. We discuss how the menu of constructs approach relates to other emerging paradigms for theory use in KT, particularly the theoretical ‘domains’ approach suggested by the work of Michie *et al*. [17]. And finally, we address some limitations and areas for future work suggested by this approach to theory building.

## Discussion

### Why study audit and feedback?

A&F is a convenient term for a heterogeneous group of interventions centered around providing feedback on existing practice to healthcare providers. A&F interventions involve the development of a summary of some aspect of clinical performance (audit) over a specific period of time, and subsequent provision of that summary (feedback) to individual practitioners, teams, or healthcare organisations. A&F has been shown to be effective in a wide variety of clinical contexts, and is one of the most commonly employed and evaluated KT interventions. However, there is enormous variability; a recent Cochrane review of 140 A&F trials showed highly variable effectiveness, ranging from substantial positive effects to null and even negative effects [25]. Such variability is at least in part due to lack of understanding of the causal mechanisms underlying A&F interventions.

The extensive A&F literature has recently been the subject of a variety of theory-guided systematic reviews, because more standard meta-analytic subgroup analyses (*e.g.*, group size, number of interventions) rarely shed light on causal mechanisms. The most recent Cochrane review reported that the effectiveness of these interventions depends in important ways on how the feedback is presented, such as the source, frequency, delivery format, and whether there is a specific target and action plan [25]. Two theory-specific reviews have targeted whether the effectiveness of A&F interventions is related to Feedback Intervention Theory [26,27] and Control Theory [28,29]. A recent review of the explicit role of theory use in A&F trials shows that relatively few trialists appear to have considered any theory during the development of their interventions [19].

These reviews show immense variability between A&F studies in terms of target audience, intervention details, targeted practice change, and context of the interventions. Without knowledge of the relevant causal mechanisms, one cannot predict whether a successful intervention will generalize, learn much from failed interventions, or successfully optimize future interventions [3]. As an analogy, studies assessing the effectiveness of new drugs would rarely be successful without considerable foundational work explicating the underlying biological mechanisms. Without similar foundational work, KT interventions such as A&F are likely to continue to be hit-and-miss propositions. In the next section, we argue that theory construction using a menu of constructs approach may have advantages over simple application of existing theories.

### Applying theories versus constructing theories using a menu of constructs

Initial work applying theory to better understand KT techniques and processes has been mostly drawn from theories of behavior from health and social psychology. For example, the Theory of Planned Behavior (TPB) describes changes in behaviors (*e.g.*, smoking cessation, changes in antibiotics prescribing habits) as being primarily determined by individuals’ intentions to engage in the behavior. Intention, in turn, is primarily determined by three factors: attitude towards the behavior, subjective norms (what important others think of the behavior), and perceived behavioral control (whether the person feels that the behavior is under their control). TPB is influential in discussions around the use of theory in KT, in part because it can be usefully applied in so many contexts (across 16 different studies of provider behaviors, these constructs correlate strongly to changes in target behavior, on average accounting for 31% of the variability [30]), in part because it is a relatively simple theory to describe and explore [3], and in part because of its ubiquity; until recently, the vast majority of theory-informed efforts to change health behaviors, particular health provider behaviors, involved versions of the TPB [21,30].

Targeting broad, generalizable theories like the TPB as an initial step towards understanding KT interventions has a lot to recommend it. Such theories focus on real-world, observable behavior as the key construct to be explained, rather than, for example, theories of human memory that are built dominantly on human performance in experimental settings and may be more difficult to generalize to non-laboratory-based settings. Simple constructs such as those comprising the TPB can be reasonably understood without an extensive disciplinary background, important in any interdisciplinary field. While constructs like ‘transfer appropriate processing’ (*i.e.*, the notion that the match between how information is encoded in memory and how it is to be retrieved will influence the likelihood its being remembered) [31] may well be relevant to many KT interventions, in its entirety it is a complex concept that is unlikely to be readily unpacked by non-specialists. The TPB specifically also comes with established methodologies for measuring the relevant constructs [32], an extremely useful criterion for content experts who may be new to the application of theory in their area.

Despite these advantages, detailed theoretical understanding of KT interventions requires investigation beyond broad theories like TPB. For example, the TPB has been criticized as a theory of KT intervention for being better at explaining intention to engage in the behavior (on average, 59% of intention is explained) than it is at explaining the behavior itself (31%) [30], for having relatively little to say about how to change and improve KT interventions that have been found to be ineffective [21], and for focusing only on voluntary human behavior, when so much of health practice and behavior has at least some automatic, rather than explicitly intentional, component [3].

To us, these general criticisms suggest a need to ‘drill down’ into specifics, to understand and describe more detailed constructs underlying the contexts, interventions, and behaviors in question. Many such constructs exist within the discipline of cognitive psychology, the scientific discipline devoted to understanding the basic mechanisms underlying human thought, including perception, memory, categorization, and judgment and decision making [33]. Many cognitive constructs seem to have face validity in the KT context and suggest specific, testable, predictions about how interventions might be made more effective. As such, they should be explored in order to examine their utility for describing and improving KT interventions.

Some work has already begun to explore the practice of combining constructs from different theories. Eccles *et al*. [34] conducted a postal survey of 230 Scottish general practitioners around management of upper respiratory tract infections without antibiotics. Noting the range of health and social psychological theories available and the lack of data on their relative merits, the study examined the extent to which constructs from a range of theories predicted hypothetical vignette-based decisions, and actual clinical behavior. Results showed that the model that explained the most variance involved constructs from multiple theories, as opposed to models restricted to an individual theory. A study looking at oral radiography behavior among 214 Scottish dental practitioners showed similar results (*i.e.*, more variance explained when using constructs from multiple theories than any individual theory on its own [35]). While Foy [36] provides a counterexample, these two studies provide intriguing initial empirical evidence to support our claim that incorporation of constructs from different theories (*i.e.*, what we are calling a menu of constructs approach) may lead to advances in understanding KT interventions.

### Examples of cognitive constructs worth exploring

We see A&F interventions as a series of mechanisms designed to improve alignment between a practitioner’s practice, the practitioner’s beliefs about his or her actual practice, and best practices as defined by the broader professional community. While all KT interventions seek to align actual practice with best practice, A&F is one of the few that also explicitly targets the fact that individual practitioners rarely have ready access to accurate information on their practice patterns [37]. A great deal of work in cognitive and educational psychology may shed light on the most effective mechanisms for enabling this alignment, but remains wholly unexplored in the KT literature. Because of the level of abstraction at which these theories were originally conceived, however, it is unlikely that any one theory will provide a complete picture of how A&F may be optimized, thus creating the need to pick and choose individual constructs from multiple theories to determine how they might apply to specific A&F contexts. Below, we present some examples of constructs that may suggest important causal mechanisms related to A&F. All of these constructs have been extensively studied, but few, if any, have been considered in the context of KT interventions. We can, therefore, offer little empirical data as to their impact on the effectiveness of A&F specifically. Instead our intent is simply to indicate how identifying such constructs can make explicit, testable predictions that can inform future research and development efforts in this applied domain.

### Two modes of reasoning

One of the most important theoretical perspectives to come out of cognitive psychology is the notion of two modes of reasoning [38,39], generically referred to as dual-processing theory. One mode, System 1 [38], can process information quickly, intuitively, and with relatively little effort. In medicine, development of knowledge structures that allow complex decisions to be made quickly is considered a cornerstone of medical expertise [40,41] and likely accounts for a great many of the decisions made during the course of any health provider’s day. The other mode, System 2, can be characterized as slow, analytic, deliberative, and effortful. Patient cases where a provider must stop to think and problem-solve invoke System 2 processing.

Some implications of this important dichotomy in human reasoning for KT have been outlined elsewhere [42,43]. A&F as a KT strategy most often invokes the System 2 mode of reasoning; information about current practice must be interpreted and understood, recommendations suggested by guidelines must be considered, and practice change implemented as deemed appropriate. The extent to which this effortful, deliberative process can affect a practice decision that is governed by System 1 processing is unclear, because the A&F literature has not generally been informed by this theoretical approach; interventions are not designed with these notions in mind, and reports do not describe interventions in these terms. However, in the context of designing A&F interventions, dual-process theory can make explicit recommendations about how to improve interventions including understanding the nature of the processing involved in the target decision, employing multi-factor interventions to target different processing modes, and considering cognitive strategies that take advantage of the strengths of both forms of reasoning [44].

### Cognitive dissonance and information discounting

Dual processing suggests that a disconnect may exist between the system targeted by A&F interventions and the system dominating actual decision-making, and that this might help explain why simple provision of feedback via an A&F task is not sufficient to ensure practice change. If this is true, one needs to ask what mechanisms might help determine whether or not the feedback is effective. One lens we can apply to this problem is the notion of cognitive dissonance [45,46], the distressing mental state that arises when we find ourselves acting in a way that is inconsistent with something we believe (*e.g.*, prescribing antibiotics, perhaps because one’s patients desire them, while believing their use should be limited) or holding two conflicting beliefs at the same time (*e.g.*, I am a good physician and my patients aren’t receiving best possible care). The state is sufficiently uncomfortable that many studies have suggested we are highly motivated to reduce the conflict experienced. The most common result in these situations is that rather than altering behavior or abandoning a belief altogether, the tension is resolved by the easier act of reinterpreting the beliefs (*e.g.*, I am a good physician and my patients are different so the guidelines don’t apply).

An important attribute of this information discounting stemming from cognitive dissonance is that it often happens without conscious awareness (*i.e.*, it resides in System 1) [47]. There is a substantial literature [48] that suggests that these sorts of automatic reinterpretations of available data are commonplace, preventing us from knowing that we are falling prey to them. Indeed, this kind of process has been proposed to be central to a generalized psychological ‘immune system’ [49] we all possess that involves automatic adaptive tendencies to rationalize in a way that enables us to maintain a sense of well-being and personal strength. These constructs clarify why, instead of accepting feedback and adjusting behavior accordingly, the result may more often be to discount the feedback itself (*e.g.*, ‘the data are not representative of my practice,’ ‘the data are biased,’ or ‘the data do not come from a credible source’). Failing to account for constructs such as these may prove a barrier to successful A&F intervention. Empowering feedback recipients to determine what data will be most relevant to their practice and how it is collected (*i.e.*, guiding the audit side of A&F) may reduce the likelihood that feedback is discounted.

### Feedback and feed forward

Work on the Feedback Intervention Theory, alluded to earlier, suggests further ways in which theoretical constructs can identify barriers that may threaten the effectiveness of an A&F intervention. For example, Feedback Intervention Theory might help explain why contextualizing feedback through displays of how the target provider compares to peers (a common strategy in A&F) can be precisely the wrong thing to do. Studies have shown that drawing attention to the recipient’s self-efficacy (*i.e.*, providing normative cues that prompt one to think specifically about where one stands relative to others along some continuum) can create a threat that makes it less likely that the feedback will have an influence [26,50]. Rather than serving as a dispassionate indicator of where improvement is possible, such feedback can invoke the psychological immune system’s defense mechanisms, again yielding cognitive dissonance and leading to the data being discounted rather than altering one’s self-assessments. Indeed, this can happen regardless of the sign of the feedback; relatively poor performance can encourage information discounting for the sake of ego defense, while relatively good performance can lead one to believe any deviations from best practice are minor and, hence, there is little to be gained from continued efforts at improvement.

Presenting data in a manner that does not create conflicting beliefs in the first place (*i.e.*, minimizes cognitive dissonance by not engendering the concern that one’s performance is substandard) may, therefore, provide an important consideration for those designing A&F interventions. Kluger and van Dijk’s [51] feed forward strategy offers an intriguing possibility that needs to be tested in an A&F context. It involves interviewing the individual about positive past experiences to help them establish an internal standard of excellence and strengthening memory traces of good performances that will influence later System 1 (similarity-based) processing, rather than focusing on the distance between the individual’s performance and an external standard. Early results suggest the procedure has the potential to enable insights into how performance can be improved without damaging self-efficacy [51].

### Desirable difficulties and cognitive load

While concerns about the amount of time and cognitive resources practitioners have available to dedicate to contemplating practice improvement leads many interventionists to design A&F interventions to be as simple and accessible as possible, this approach does not consider the clear gains to be derived from requiring the right kind of effort on the part of the target provider. Bjork has put forward the notion of desirable difficulties [52-54], which suggests that we are better able to learn, remember, and make use of information when we are put in situations that induce errors. These models are aimed deliberately at helping people overcome the false perception that they have understood and learned material in a way that will enable its use in the future. For example, being tested on material has robustly been shown to yield better learning than studying the same material multiple times, even though study often yields feelings of fluency that we erroneously infer to indicate that learning has taken place [55]. Testing (*i.e.*, being required to effortfully retrieve information from memory), increases the likelihood that we will be able to retrieve the information from memory again in the future and makes it more likely that our attention will be productively focused on areas of knowledge that require further study [55]. If implemented well, such desirable difficulties might also improve cognitive dissonance by making it more difficult to discount information that one has exerted effort to collect.

One can further specify this issue in terms of cognitive load, a notion that requires us to consider the various types of load that can impact on our mental processing capacity. Intrinsic load (the amount of information to be learned), extraneous load (created by the way in which the information is presented), and germane load (the resources required of working memory to deal with intrinsic and extraneous load) are believed to play different roles in learning. Too many competing factors (*e.g.*, having to read through pages of text in a busy clinical environment to understand the feedback provided by an audit) create too much extraneous load and suboptimal learning. Presenting material with a degree of difficulty greater than the learner is prepared to process can similarly increase intrinsic load to the point of disadvantage [56]. By contrast, too little germane load (*i.e.*, not engaging working memory to a great enough extent) can create a situation where feedback recipients passively accept information, but are not convinced sufficiently (or prompted to elaborate on the information enough) for the learning to have a long-term impact. Considerable research, largely focused on designing multimedia learning platforms, has demonstrated principles that can optimize these various kinds of load [57-59]. Consideration of these principles in light of A&F interventions may be a fruitful area of research.

### Communities of practice and adaptive expertise

While the examples used to this point have largely focused on the psychology of the individual learner, A&F interventions are often targeted at teams or practices rather than individuals [60-65]. The notion of Communities of Practice [66] describes that social networks of individuals who share a concern and interact regularly around that topic offer substantial benefits for learning. The three crucial characteristics, according to Wenger, are a clearly identifiable domain (*i.e.*, an area of shared competence that distinguishes members of the community from others), the community itself (with relationships nurtured to support and help one another in the group’s joint activity), and practice (*i.e.*, activity oriented around a shared repertoire of experience, expertise, and resources). Practicing in isolation has been found to be a main predictor of underperformance [67]. Such Communities of Practice might be thought of as informal, ongoing A&F opportunities, and suggest ways in which relationship-centered education [47] and mentoring can be incorporated with opportunities for desirable difficulties and reduced cognitive dissonance to develop novel models of A&F.

These models remind us of theoretical notions of adaptive expertise [68,69], which suggest that expertise should not be conceived of as a ‘thing’ that can be achieved, but rather, should be considered an approach to continuous efforts at quality improvement. Engaging in this way requires a reward structure that the respect, nurturing, and collegiality of one’s peers can create. Individuals need to feel safe in discussing their clinical practice, but at the same time, simply conveying information to them with no opportunity to discuss the issues with others and come to some mutual understanding of how to alter performance appropriately increases the likelihood that one may inappropriately discount external data that should not be discounted. At the end of the day, these models, when combined with various political and economic theories, suggest that establishing a system whereby the reward structure for healthcare providers encourages deliberate effort to engage with A&F may prove particularly influential in ensuring their effectiveness.

## Summary

Our examples of theoretical constructs derived from cognitive and educational psychology all have one thing in common. They all stem from theories that, because of the context and/or level of abstraction at which they were originally developed, could not hope to provide anything like a full picture of an A&F intervention. As a result, these theories would generally not be considered relevant to KT interventions, because the context for which they were developed is so far removed from complex A&F interventions. Nevertheless, they do provide specific, testable hypotheses about ways in which A&F interventions might be improved. Thus the need for adopting a menu of constructs approach; each theoretical construct mentioned (and the many more that were not mentioned) promise productive lines of inquiry that can yield greater guidance regarding how to adapt A&F strategies to particular settings and how to productively and efficiently test the effectiveness of such strategies.

### Menu of constructs and the theoretical domains framework

This menu of constructs approach is not inconsistent with an important new approach to the use of theory in KT research started by Michie *et al.*[17]. Noting the number of health and social psychological theories potentially relevant to such research, as well as the considerable overlap in constructs among them, this group engaged in a consensus process that distilled from 33 different theories a set of 12 behavior change ‘domains’ agreed to be relevant to implementation research. These domains are intended to be distillations of different, but related, constructs from different theories, ones that nevertheless have common implications for behavior change and implementation research. This offers an example of how the menu of constructs approach might be implemented. By beginning from these 12 domains (now updated to 13) [70], this Theoretical Domains Framework (TDF) offers a systematic means to consider a wide range of theoretical approaches, and to narrow one’s search for theories relevant to a specific KT context.

The TDF has since been extended in at least two different ways. First, it has been directly employed to recommend specific implementation intervention techniques, based on expert agreement on what intervention techniques are implied by each domain [71,72]. Such an approach can provide concrete guidance for interventionists, but may do so without explicit reference to specific theories or constructs.

A second approach, referred to as Theoretical Domains Interviewing (TDI) [73,74], involves developing an intervention by interviewing or surveying the target audience and using the domains to prompt participants to identify barriers and facilitators to the target behavior. Responses are then categorized into the most relevant theory-specific constructs, and an intervention is developed based on the recommendations of the theory with the most constructs identified as being relevant to the target behavior. TDI therefore provides a systematic approach to identifying which theory may be most appropriate to the new context.

This second approach to applying the TDF specifically seeks to select ‘the most appropriate theories to develop interventions for changing specific behaviors’ [73]. Because the goal is to identify what is relevant at the level of the theory rather than the level of the construct, however, the approach might fail to target constructs such as those discussed in this paper. Because theories from cognitive psychology often seek to explain mechanisms rather than behaviors (*i.e.*, they explain at a lower level of abstraction), they may not, in isolation, lend themselves to explaining complex behaviors and, as such, exclusive use of TDI may lead us back to the issues that began this paper (*i.e.*, those that arise when individual theories are adopted in an all-or-none manner). This is likely the reason why the original TDF [17] combined much of cognitive psychology into a single construct (memory, attention, and decision processes); because of the level of abstraction problem, it is not clear how the many theories within cognitive psychology might be relevant to implementation. It is only at the level of the construct that it becomes clear how such processes can inform KT interventions.

We propose a simple fix to marry the TDI with the menu of constructs approach that will simultaneously indicate how the menu of constructs approach might be productively implemented. Rather than identifying constructs in the interviews as a means to identifying the most relevant theory, in some cases it might be worthwhile to consider all constructs deemed relevant in the interviews, and use them to construct a new theory specific to that KT context. This approach would allow development of interventions and theory that incorporates constructs from various theories. Such an approach would be consistent with our menu of constructs idea, allow for the incorporation of constructs at different levels of abstraction, and also make use of the important methodological advances from the TDF and TDI.

We believe this approach would help overcome some thinkers’ objections to the utility of using theory in KT research [9,10]. Use of theory need not be in opposition to detailed empirical understanding, but instead should serve as an orienting conceptual framework that can be used iteratively to both guide and to be influenced by empirical observation. Much of the negative connotation that can be associated with the word ‘theory’ comes from confounding of the term with high level conceptualizations that may describe a problem well, but offer little in the way of concrete guidance regarding specific mechanisms whereby practice can be changed. Theory that is so broad as to be applicable to any situation may inevitably be so weak as to yield little more than adages that frame an outcome after it has occurred. For example, theories that conceive of creativity as a tendency to ‘think outside the box’ can provide adequate descriptions of activities that define creativity, but ultimately offer little guidance regarding how to do so effectively.

The most effective methods for implementing this menu of constructs approach have yet to be established. We plan to present experts in a wide range of theoretical domains with example A&F interventions, eliciting their opinions about theories and constructs that they feel make testable predictions about how to improve the interventions. Once a laundry list of constructs is assembled, we anticipate conducting pilot evaluations in the form of written vignettes, usability testing sessions, or small scale randomized controlled trials to assess which candidate constructs warrant more formal evaluation. This pilot testing process could also incorporate the results of bottom-up analyses of the target behavior and context, of the sort recommended by others [75].

### Limitations

This menu of constructs approach may be seen to have much in common with Bandura’s pejorative term ‘cafeteria-style research’ [76]. He argues that picking constructs from various theories and recombining them can lead to needless proliferation of essentially identical constructs with different names. When one’s goal is integration of multiple overlapping theories into one all-encompassing theory, such proliferation is clearly a problem. However, we see the process of theory development in KT as being distant from such a grand unifying theory. In the context of A&F interventions (only one of many possible KT interventions), we are only beginning to understand what factors predict an effective intervention. What is becoming clear is that the broad social cognitive constructs such as those offered by the TPB have not offered a sufficiently detailed theoretical description to help us to consistently design effective A&F interventions, and that the door must be opened to theory from a broader range of disciplines to understand these complex interventions [77]. When higher-order models do not provide sufficient help, ‘drilling down’ to more complex and context-specific aspects of behavior seems only sensible.

Another potential limitation of the menu of constructs approach is that by incorporating individual constructs independently of the models with which they were developed, one may lose some of the power of the original theory, and potentially some of the meaning of the construct itself. Theories are comprised not only of constructs, but of the proposed relationships between constructs as well. Porting constructs into new contexts, separate from these relationships, may have unexpected implications for the utility of the construct. For example, will the ‘perceived behavioral control’ construct from the TPB have the same explanatory value independently of the other TPB constructs? We believe that this problem is one of validation. No one is suggesting that all potentially relevant constructs will prove useful in every context. Rather, adoption of a menu of constructs approach is meant to offer specific prompts that necessitate the validation of each construct within the context of the new theory being built. In the newly resulting theoretical context, any individual construct may or may not add explanatory value, and cannot therefore be included solely based on its utility in its original theory.

Finally, while in general we see the flexibility inherent in the menu of constructs approach to be a way forward in the KT literature, that flexibility may sometimes be more of a hindrance than a help. One of the attractions of the TDF approach is that it boils down many theories into a few key domains, which may be seen as more tractable from the point of view of designing implementation strategies. In contrast, the menu of constructs approach widens the number of constructs to consider even further, by incorporating theories that to date have not been considered in a KT context. We feel that different tools will suit different purposes, and that further work attempting to use the menu of constructs approach will allow us to fruitfully explore these issues in more detail.

As with any component of evidence-based medicine, theory should be applied judiciously rather than adopted lock, stock, and barrel with no consideration of the individual idiosyncrasies created by different contexts. Furthermore, as KT researchers, the goal of theory building should remain firmly on how to create more effective interventions. If the menu of constructs approach allows us to develop a better understanding of the range of theories from multiple disciplines available to us, and to engage in systematic study of the applicability of their constructs, we will be better positioned to make clear recommendations about how to implement newly developed knowledge more effectively.

## Competing interests

The authors declare that they have no competing interests.

## Authors’ contributions

Both authors contributed to all aspects of the manuscript. Both authors read and approved the final manuscript.
